# Supplementary material for: Neuronal parts list and wiring diagram for a visual system
Source: Nature. 2024 Oct 2;634(8032):166–80. doi: 10.1038/s41586-024-07981-1 (PMC11446827; doi:10.1038/s41586-024-07981-1)
Supplement: Supplementary file 5 — Cards for all cell types. Each card has 6 panels (clockwise): logical connectivity predicate diagram, numeric stats, illustration of a typical cell, followed by 3D rendering of all cells from three orthogonal viewpoints. Also available at Codex. [file 41586_2024_7981_MOESM5_ESM.zip › pdf/Y1.pdf]

# Y1

predicate precision: 59%, recall: 98%

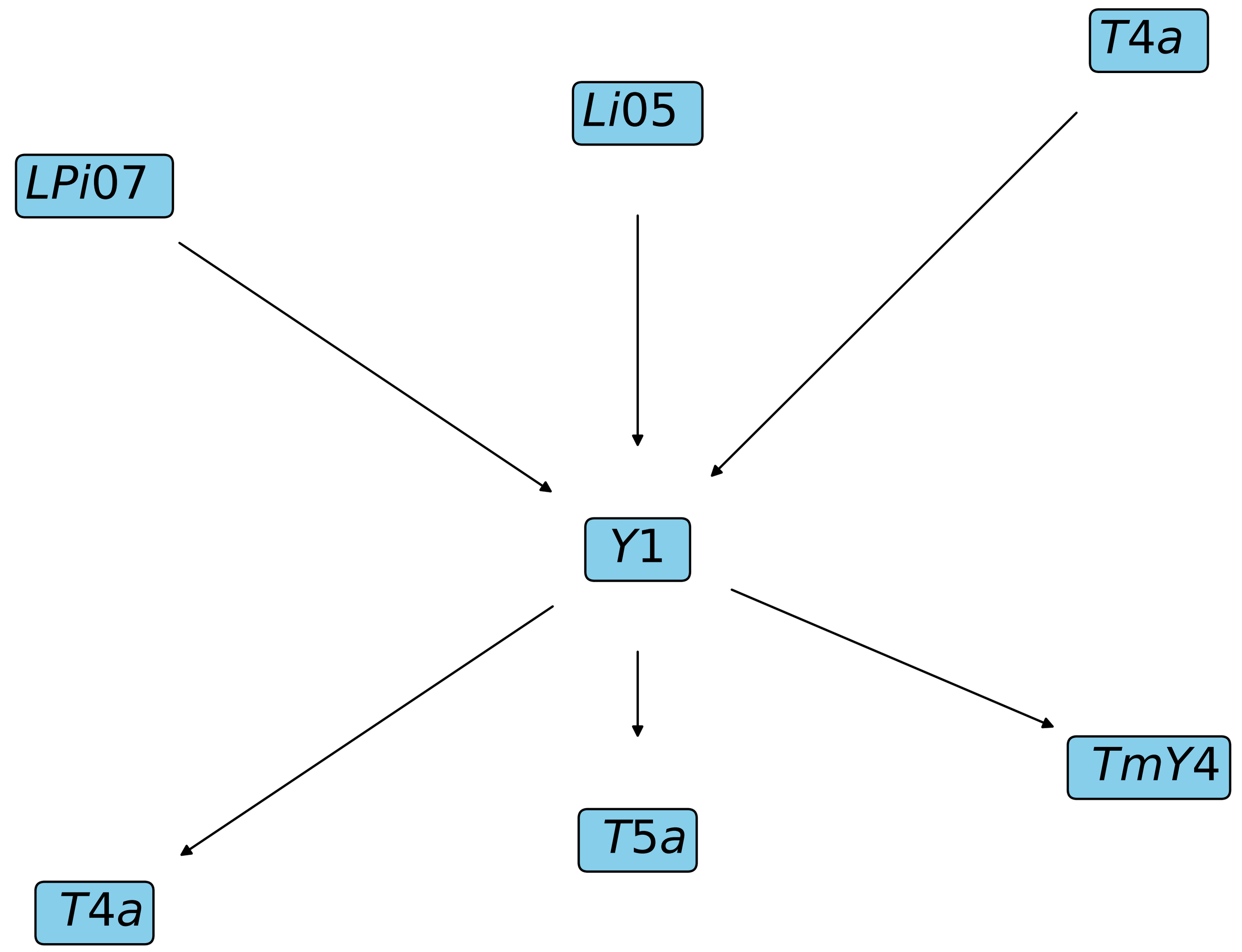


avg. stats

| | |
|---|---|
| cells | 82 |
| NT prediction | GLUT (99%) |
| top in/out regions | LOP (77%) / LOP (66%) |
| in/out degree | 106 / 115 |
| in/out synapses | 501 / 516 |
| cable len | $1,133\mu$ |
| surface | $1,940\mu^2$ |
| volume | $133\mu^3$ |

morphology

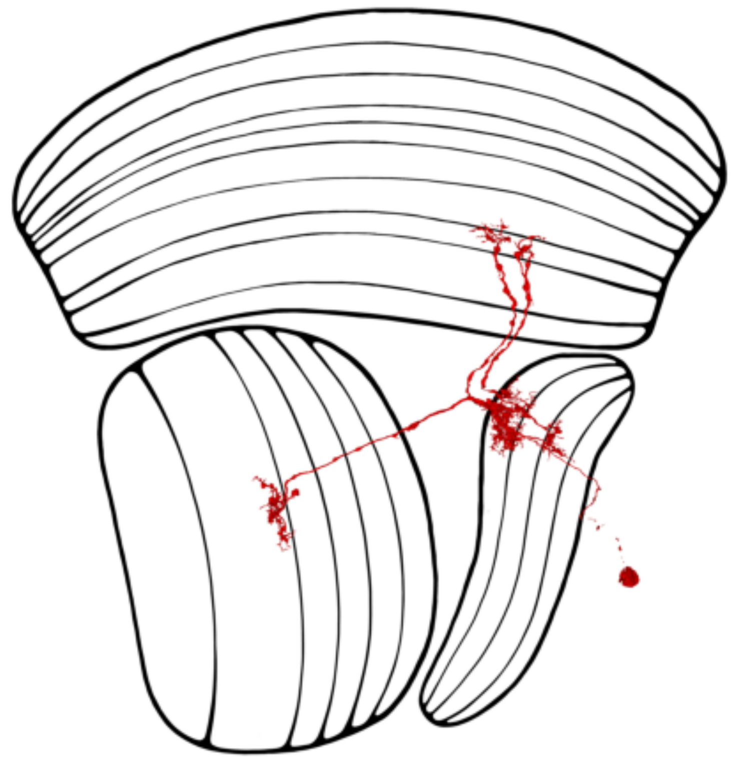

anterior view

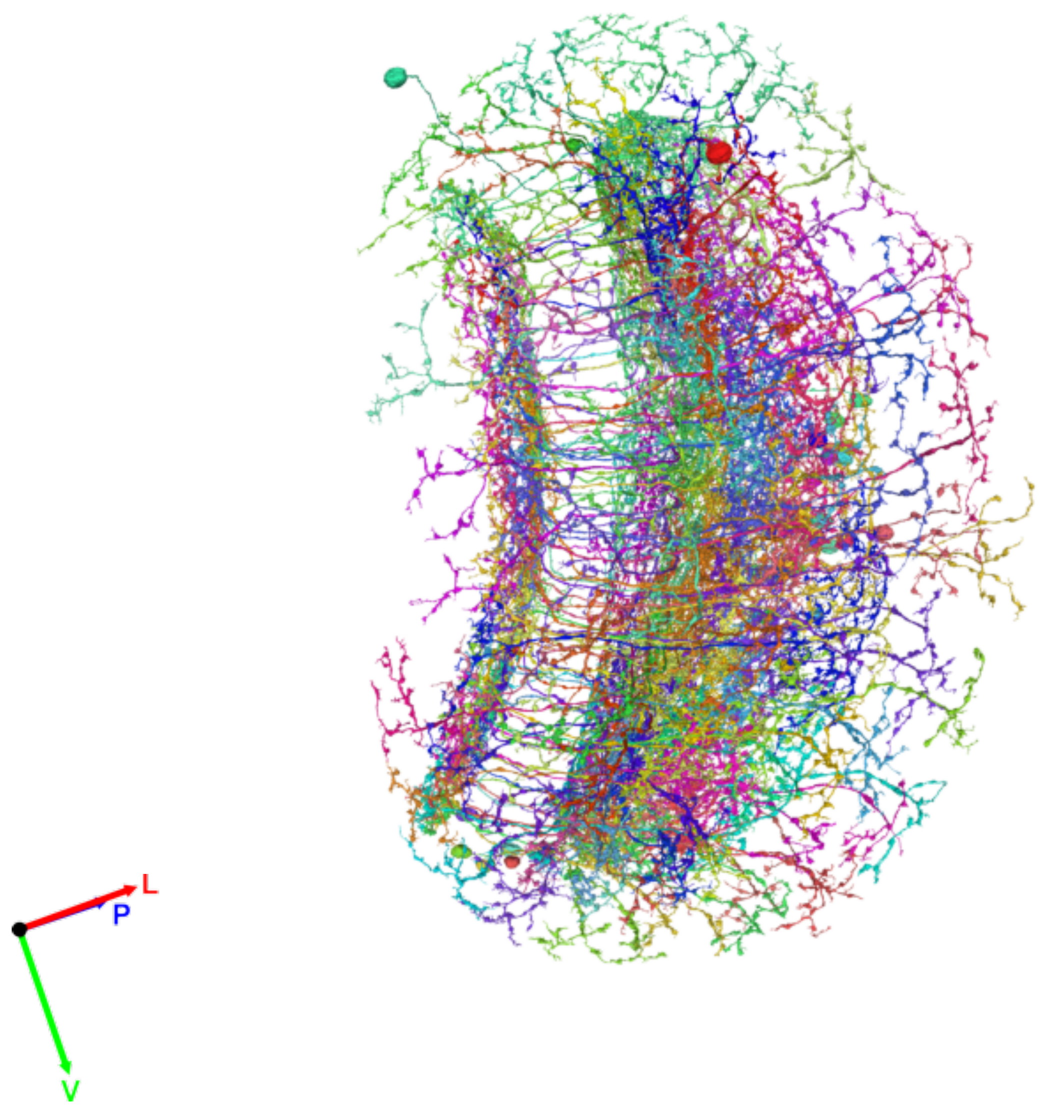


ventral view

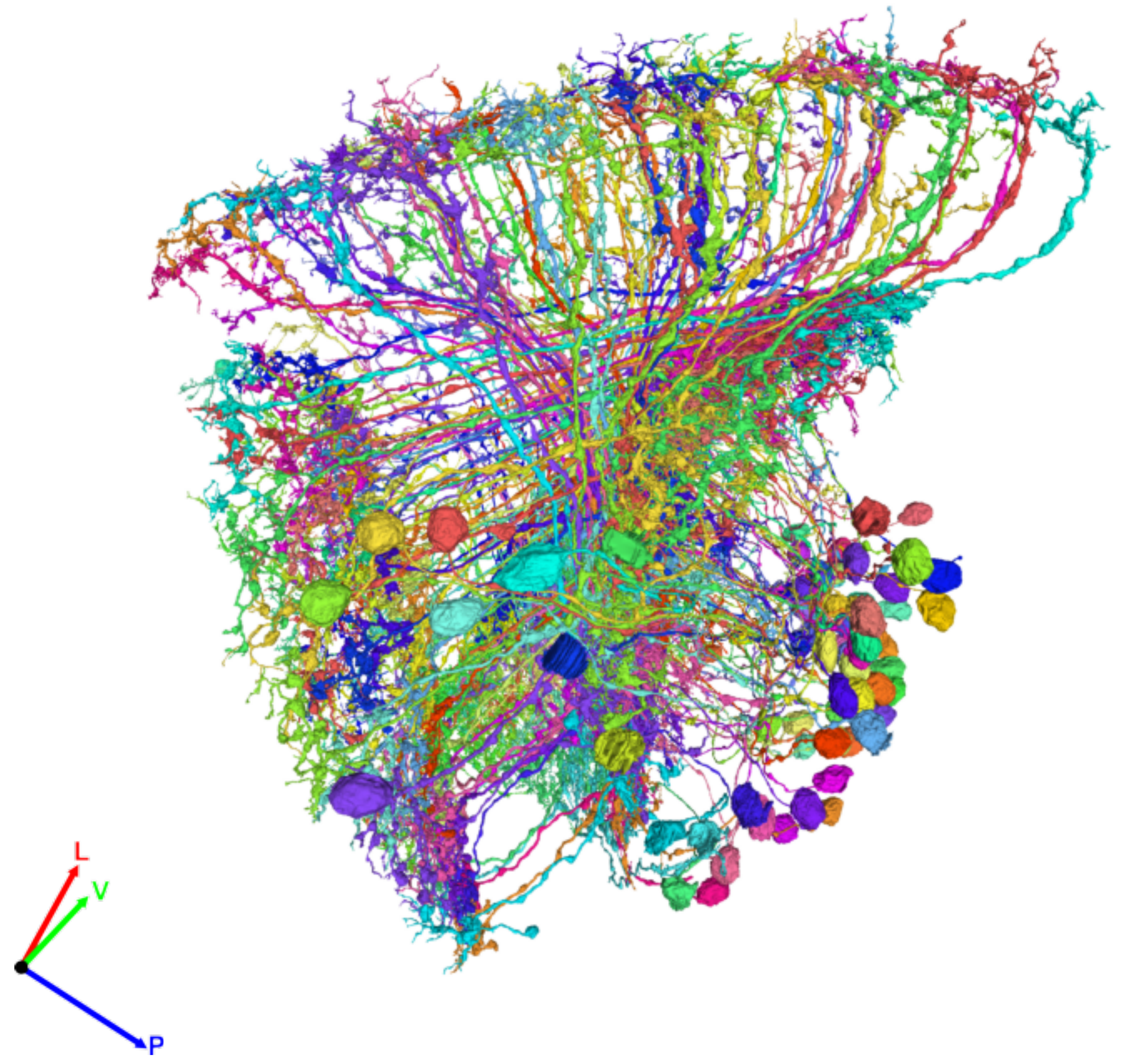


side view

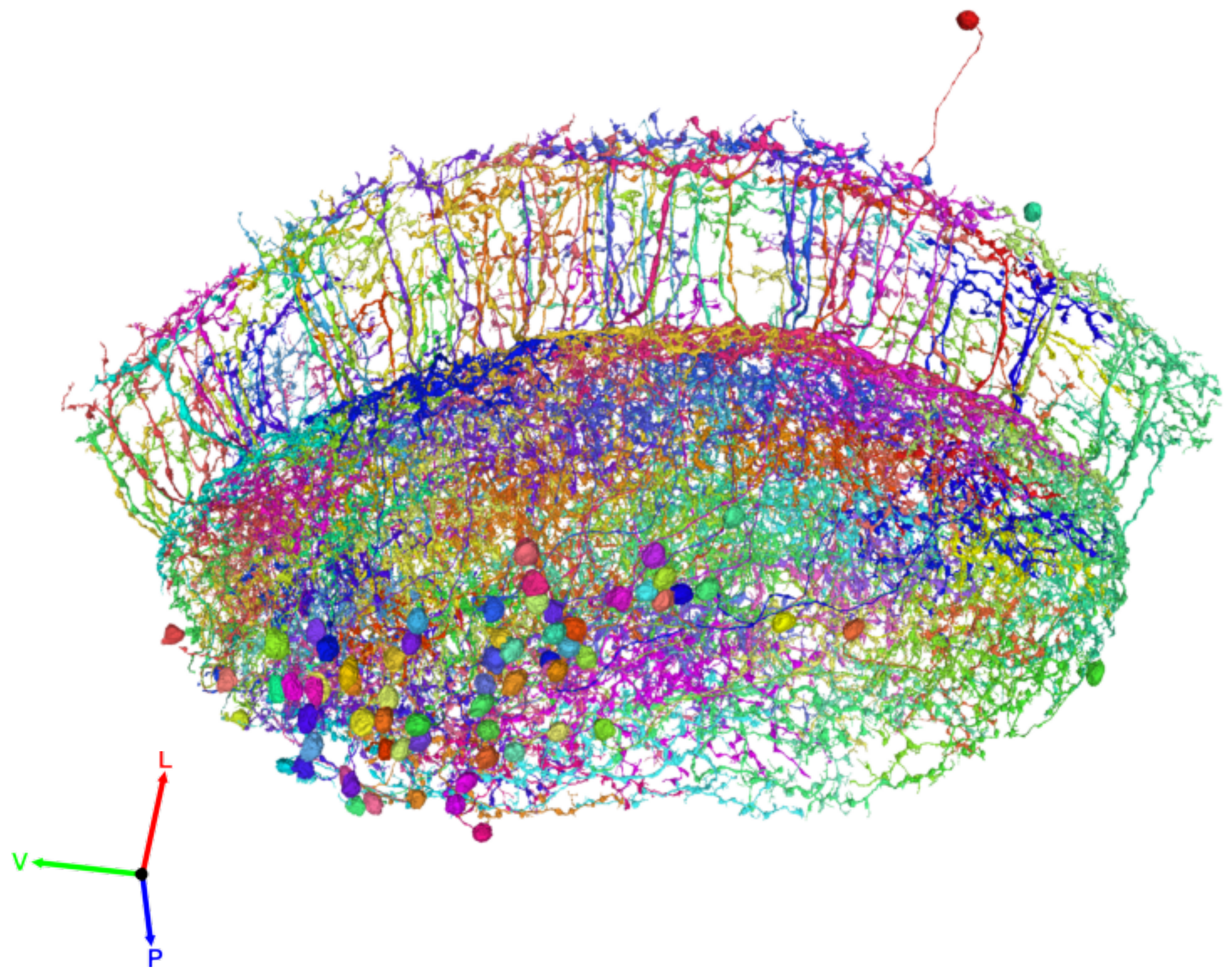


Y Neuron
